# Monitoring and Risk Assessment of Pesticide Residues in Fishery Products Using GC–MS/MS in South Korea

**DOI:** 10.3390/toxics12040299

**Published:** 2024-04-18

**Authors:** Myungheon Kim, Mihyun Cho, Seo-Hong Kim, Yoonmi Lee, Mi-Ra Jo, Yong-Sun Moon, Moo-Hyeog Im

**Affiliations:** 1 Department of Food Engineering, Daegu University, Gyeongsan 38453, Republic of Korea; 2 Department of Environmental and Biological Chemistry, Chungbuk National University, Gyeong 28644, Republic of Korea; 3 Food Safety and Processing Research Division, National Institute Fisheries Science, Busan 460083, Republic of Korea; 4 Department of Horticulture and Life Science, Yeungnam University, Gyeongsan 38541, Republic of Korea

**Keywords:** fishery products, pesticide residues, risk assessment, monitoring, GC-MS/MS

## Abstract

The aim of this study was to assess the risk of pesticide contamination in aquaculture and its impact on fishery products. We conducted an assessment of 300 samples collected from nine regions in South Korea, including various types of seafood, such as freshwater fish, marine fish, crustaceans, and shellfish. Pesticide residues in seafood were analyzed using GC–MS/MS after sample preparation using a modified QuEChERS method, revealing the presence of eight pesticides (4,4′-DDE, 4,4′-DDT, boscalid, isoprothiolane, oxadiazon, pendimethalin, thifluzamide, and trifluralin) across seven fish species (carp, far eastern catfish, crucian carp, eel, Chinese muddy loach, mirror carp, and sea bass). Following the grouping of DDE with DDT, a risk assessment of fishery products was conducted. After the estimated daily intake (EDI) of fish was calculated and compared with the acceptable daily intake (ADI), the health risk index (HI, %ADI) of the detected pesticides was evaluated and found to be 1.07% or lower. The results suggest that the consumption of domestically farmed fish products in South Korea poses minimal health risks associated with pesticide residues.

## 1. Introduction

Pesticides, which are chemicals used to control pests that damage crops and regulate physiological functions (growth, ripening, etc.), provide various benefits, including increased agricultural production, improved quality, and reduced production costs. However, most pesticides, primarily synthetic organic compounds, are toxic upon entering the human body, leading to disruptions in the nervous and enzyme systems. Therefore, careful management of pesticide usage is necessary. Furthermore, the direct application of powdered and granular pesticides to soil may result in runoff during rainfall due to improper disposal methods, solubility, and octanol/water partition coefficients (log Kow) [1,2,3,4]. Consequently, these pesticides may persist in water and enter streams near agricultural areas, causing water pollution in rivers and oceans and ecological issues such as bioaccumulation in aquatic species inhabiting the watershed, potentially causing reproductive failure [5,6].

To address these concerns, various countries, including South Korea [7], the United States [8], Australia [9], the European Union [10], and Japan [11], have established maximum residue limits (MRL) to regulate pesticide residues in domestically produced and imported fish and other food products. The Codex Alimentarius Commission (Codex) establishes international standards to ensure fairness in global food trade [12].

Seafood consumption in South Korea is relatively high on a global scale [13]. According to the Food and Agriculture Organization of the United Nations (FAO) in 2021, Portugal and South Korea showed the highest annual seafood consumption per capita, at 59.4 and 55.6 kg, respectively, surpassing Japan (45.1 kg), Spain (40.1 kg), and China (39.9 kg) [14]. As aquaculture production and seafood imports continue to rise, it is imperative to establish a systematic monitoring system to detect residues in both domestic and imported seafood.

Previous studies have detected several pesticides, including isoprothiolane, hexaconazole, diazinon, chlorpyrifos, prothiofos, alachlor, butachlor, and molinate, at levels ranging from 0.027 to 12.871 ng/g, in six major river basins in South Korea. An ecological risk assessment of three aquatic species revealed that these pesticides did not exceed the hazard quotient indexes of 1.0, suggesting no potential harm to the aquatic ecosystem [15]. Additionally, contamination levels of organochlorine pesticides were examined in edible marine organisms, including olive flounder, soft shell clam, thread-sail filefish, and abyssal searobin, near Busan Yongho Port, revealing residue levels of dichlorodiphenyltrichloroethane (DDT) and hexachlorocyclohexane (HCH) within safe limits for humans according to the US EPA criteria (both chronic and acute evaluations) [16].

Hydrophobic organochlorine pesticides, such as DDT, were extensively used in the past but have been banned or restricted in developed countries since the 1970s owing to their severe human and environmental toxicity and persistence [17]. However, their past use and their current use in some countries have resulted in the migration and bioaccumulation of these substances in various organisms, including fish. As a result, aldrin, chlordane, DDT, dieldrin, endrin, heptachlor, hexachlorobenzene, and mirex have been listed as persistent organic pollutants (POPs) under the Stockholm Convention since 2001 [18].

Extensive research has been conducted internationally to ensure the hygiene and safety of seafood. Benzene hexachloride (BHC), DDT, and dieldrin are routinely monitored in seafood products in the United States, Europe, Australia, and Canada [19]. Additionally, the U.S. Food and Drug Administration (FDA) conducts an annual national fish and shellfish sanitation program [20], and the Environmental Protection Agency (EPA) monitors fish populations in the inland water and estuaries of the country for an extended period of time. The National Marine Fisheries Service (NMFS), a division of the National Oceanic and Atmospheric Administration (NOAA), employs an inspection system to ensure quality standards and enforce regulations on fishery-processed products [21].

Despite recent efforts to enhance safety management in South Korea through the introduction of a positive list system (PLS) [22], MRL for 447 pesticides, excluding ethoxyquin, have not yet been established for aquatic products. Therefore, it is crucial to collect extensive monitoring data on contaminated foodstuffs and residual substances. Particularly in aquaculture, measures must be established to manage pesticide components in seafood through risk assessments based on residue investigations to address the potential risks of unintended contamination from terrestrial sources into fish farms.

This study investigated the residue levels of 44 pesticides, including DDT, BHC, and boscalid, using GC–MS/MS in a total of 300 samples from the market, including 20 samples each of seven species of freshwater fish, six species of marine fish, one species of crustaceans, and shellfish. The potential health risks associated with the estimated pesticide intake from seafood were subsequently evaluated.

## 2. Materials and Methods

### 2.1. Chemicals and Materials

The 44 pesticides, including 2,4′-DDD, 2,4′-DDE, 2,4′-DDT, 4,4′-DDD, 4,4′-DDE, 4,4′-DDT, alachlor, aldrin, ametryn, atrazine, boscalid, buprofezin, carfentrazone-ethyl, chlordane-cis, chlordane-trans, chlorothalonil, chlorpyrifos, cypermethrin, deltamethrin, dieldrin, α-endosulfan, β-endosulfan, endosulfan sulfate, endrin, fenitrothion, heptachlor, heptachlor epoxide-cis, heptachlor epoxide-trans, hexachlorobenzene, iprobenfos, isoprothiolane, mirex, oxadiazon, pendimethalin, permethrin, prometryn, tebuconazole, terbutryn, tetraconazole, thifluzamide, trifluralin, α-HCH, β-HCH, and γ-HCH, were purchased from Kemidas (Gunpo, Republic of Korea). HPLC-grade solvents, namely acetonitrile and hexane, were acquired from J. T. Baker (Centre Valley, PA, USA), and dichloromethane was obtained from Honeywell (Charlotte, NC, USA). Conical tubes (50 mL) were purchased from SPL (Pochoen, Republic of Korea), and centrifugation was performed using Megafuge 1.R (Thermo Fisher Scientific, Waltham, MA, USA). Acetic acid (≥99.7%) and MgSO_4_ (99.5%) were sourced from Sigma–Aldrich (St. Louis, MO, USA), whereas NaAc (98.5%) was purchased from Junsei (Tokyo, Japan). Florisil cartridges for purification were obtained from Sep-Pak Vac 6cc (500 mg) (Waters, Wexford, Ireland).

### 2.2. Sampling Procedures and Sample Preparation

Seafood samples were purchased from seafood markets in nine regions nationwide (Gyeonggi-do, Gangwon-do, Gyeongsangnam-do, Gyeongsangbuk-do, Jeollanam-do, Jeollabuk-do, Chungcheongnam-do, Chungcheongbuk-do, and Jeju-do). The seafood samples were all from aquaculture in South Korea. A total of 300 samples (20 samples per species) were obtained in accordance with the seafood sampling criteria of the Food Code. Sampling encompassed freshwater fish (carp, far eastern catfish, crucian carp, eel, Chinese muddy loach, mirror carp, and rainbow trout), marine fish (olive flounder, flathead mullet, red seabream, starry flounder, Korean rockfish, and sea bass), crustaceans (whiteleg shrimp), and shellfish (abalone) [23]. For sample preparation, whiteleg shrimp (with shell and viscera removed) and fish (with fishbone, fins, and head removed) were homogenized in a mixer (Grinmic gold-DA10000G, Daesung Artlon, Paju, Republic of Korea) using dry ice to grind the edible parts, including the skin. Samples were then stored in a freezer at −20 °C until analysis.

### 2.3. Residual Pesticides Analysis

The analysis of the target pesticides was conducted using a modified Pesticide Analytical Manual (PAM) method and Association of Official Agricultural Chemists (AOAC) protocol [24] based on the quick, easy, cheap, effective, rugged, and safe QuEChERS method used for multi-component analysis [25].

Sample preparation was performed as follows: 300 g (minimum) of the edible portion was ground, and precisely 5 g was transferred to a 50 mL conical tube with 20 mL of acetonitrile containing 0.1% acetic acid, and the mixture was shaken at 2000 rpm for 20 min. Subsequently, 4 g of magnesium sulfate (MgSO_4_) and 1.5 g of sodium acetate (NaOAc) were added to the tube, which was shaken again at 2000 rpm for 5 min, followed by centrifugation at 4000× *g* (4 °C) for 10 min. The supernatant (4 mL) was transferred onto a conical tube containing 600 mg of MgSO_4_ and centrifuged (4000× *g* (4 °C) for 10 min). Of this, 2.5 mL of the supernatant was concentrated under rotary evaporation (30 °C), and the residue was dissolved in 2.5 mL of hexane. Two milliliters of the hexane solution was loaded onto a Florisil cartridge pre-activated with 5 mL of hexane flowing at a rate of 2–3 drops/s and collected in a test tube. Then, 5 mL of a mixture of dichloromethane, acetonitrile, and hexane (50.0:3.5:46.5, *v*/*v*/*v*) was eluted in 1 mL portions and collected in the same test tube. After the eluted solutions were concentrated under nitrogen at 40 °C, they were dissolved in 1 mL of hexane and mixed well using a vortex mixer for GC–MS/MS analysis. Each sample was analyzed in triplicate, and the mean value was calculated.

### 2.4. Instruments and Analytical Conditions

Pesticides were simultaneously analyzed using an 8890 GC apparatus combined with a 7010B MS instrument from Agilent Technology (Santa Clara, CA, USA). The GC apparatus was equipped with a DB-5 ms column (30 m length × 0.25 mm inner diameter × 0.25 μm film thickness; Agilent Technology, Santa Clara, CA, USA). Data were processed using MassHunter Quantitative Analysis software (Agilent, Santa Clara, CA, USA). The oven temperature was initially maintained at 60 °C for 0.2 min, and then it was increased to 180 °C (ramping rate of 20 °C/min) and to 250 °C (hold 3 min) at a rate of 15 °C/min. Thereafter, the oven temperature was increased to 300 °C (ramping rate of 20 °C/min), which was maintained for 5 min. Helium was used as the carrier gas, and its flow rate was 1.2 mL/min. The injector temperature and injection volume were 260 °C and 1 μL in split mode (5:1), respectively. The temperature of the ion source was 250 °C, and ions were obtained in the multiple reaction monitoring mode at an electron ionization of 70 eV. Two precursor ions paired with two product ions were quantified and qualified. The *m*/*z* values and collision energies of the precursor and product ions for each pesticide are shown in Table 1.

### 2.5. Method Validation

The analytical methods used to analyze pesticide residues in seafood products were validated through linearity, limit of detection (LOD), limit of quantification (LOQ), recovery, and repeatability in accordance with the guidelines outlined in the manual of the Codex Alimentarius Commission (CAC, 2023) [26].

For the validation of the method, representative species of each fishery product were selected. These included eel (fatty fish) from freshwater sources, olive flounder from saltwater sources, whiteleg shrimp from the crustacean category, and abalone from the shellfish category. Pesticide-free samples were used as the control during the validation of the method. Linearity was determined using matrix-matched calibration, and the coefficient of determination (R^2^) was calculated. LOD and LOQ were calculated using the signal-to-noise (S/N) ratio of chromatographic peaks from analyses of pesticide-free samples, which were set differently for each pesticide component. When an S/N ratio of 3 or higher was designated as the LOD, the LOQ was defined as an S/N of 10 or higher. The recovery test for accuracy and precision of methods was repeated 5 times after adding mixed standard solutions at 1×, 10×, or 50× LOQ levels.

### 2.6. Risk Assessment

Risk assessment is the prediction of potential harmful effects and the probability of their occurrence when the human body is exposed to hazardous substances present in food or other sources. Risk can be evaluated by quantitative and qualitative calculation of the amount or levels of hazardous substances ingested [27]. Exposure assessment was conducted by calculating the estimated daily intake (EDI) using 9 scenarios (Table 2) based on food intake and the detected amounts in this monitoring study. Consumption data of the target species were extracted from the average and extreme (99th percentile) intake of fish and shellfish over a 5-year period (2017–2021) from the national health statistics of Korea National Health and Nutrition Examination Survey (KNHANES) [28]. The average body weight of Koreans was assumed to be 60 kg. In the absence of an intake value for the monitored species, it was calculated as 1/2 of the minimum mean and extreme (99th percenile) intake of the species in each category.

Seafood was classified into three categories—fish, crustaceans, and shellfish—which included the monitored species. The average consumption amounts for all fish species (21 species in 2017, 17 in 2018, 16 in 2019, 14 in 2020, and 13 in 2021), crustaceans (2 species in 2017–2021), and shellfish (6 species in 2017, 7 in 2018, 7 in 2019, 5 in 2020, and 5 in 2021) were combined for each respective year, and the average value was used as the consumption amount for each group. Additionally, the detection of pesticide concentrations was based on the results obtained from this monitoring process.

Estimated daily intake (EDI) was calculated by the equation below.

(1) EDI(ng/person/day)=DFI(g/person/day)×DPC(ng/g)

The health risk index (HI, %ADI) was calculated by comparing the calculated EDI to the acceptable daily intake (ADI) and expressed as a percentage of the ADI.

(2) HI(%ADI)=EDI(ng/person/day)/ADI(ng/person/day)×100

### 2.7. Data Analysis

Data analysis was performed using Microsoft Office Excel 2013 (Microsoft Corporation, Seattle, WA, USA). The mean and standard deviation (SD) values were calculated from five replicates.

## 3. Results and Discussion

### 3.1. Method Validation

The LOQ for the majority of tested pesticides ranged from 7 to 9 ng/g, whereas lipophilic pesticides (chlorothalonil, cypermethrin, deltamethrin, dieldrin, hexachlorobenzene, permethrin, prometryn, and tebuconazole) exhibited an LOQ of 10 ng/g (Table 3). A standard calibration curve was used to verify linearity through GC–MS/MS analysis of the matrix-matched calibration curve according to the LOQ. The concentrations for the standard calibration curve for the pesticides were prepared differently depending on the LOQ values: 1.4, 3.5, 4.9, 7, 8.4, 10.5, and 14 ng/g for 7 ng/g; 1.6, 4, 5.6, 8, 9.6, 12, and 16 ng/g for 8 ng/g; 1.8, 4.5, 6.3, 9, 10.8, 13.5, and 18 ng/g for 9 ng/g; and 2, 5, 7, 10, 12, 15, and 20 ng/g for an LOQ of 10 ng/g. The coefficient of determination for the standard calibration curves of the 44 pesticides ranged from 0.99384 to 0.99947, complying with the Codex recommended guideline (R^2^ > 0.98), which indicated excellent linearity. Therefore, this analytical method was suitable for quantitative analysis during monitoring. Furthermore, the recovery rate test for the 44 pesticides on the representative species showed values ranging from 70.0% to 117.8% across all concentrations, meeting the criteria required for validating the analysis method according to the Codex guideline (70–120%).

### 3.2. Monitoring Results of Pesticide Residues in Seafood

The residue concentrations of the 44 pesticides were analyzed in 300 samples, comprising 20 of each of 15 seafood species collected from nine regions nationwide (Table 4). Out of the 300 samples, 27 cases were detected in seven fish species; this included eight different pesticides, with 18 cases found in freshwater fish and 9 cases in marine fish out of 140 and 120 samples, respectively. Freshwater mirror carp exhibited the highest number of various pesticides, including boscalid, isoprothiolane, oxadiazon, and trifluralin. Boscalid was detected in two cases at 8 ng/g each, whereas isoprothiolane was found in one case at 8 ng/g. Oxadiazon was detected in two cases at 8 ng/g and 10 ng/g, and trifluralin was found in one case at 7 ng/g. In sea bass, three pesticides (4,4′-DDE, 4,4-DDT, and pendimethalin) were detected at levels of 10 ng/g, with six cases of 4,4′-DDE, two cases of 4,4′-DDT, and one case of pendimethalin. In crucian carp, two pesticides were detected, one case of oxadiazon (7 ng/g) and two of thifluzamide (10 ng/g). Similarly, two pesticides (4,4′-DDE, pendimethalin) were detected in the Chinese muddy loach, with three cases of 4,4’-DDE (7–8 ng/g) and one of pendimethalin (7 ng/g). Only one pesticide was found in carp (4,4′-DDE, one case at 7 ng/g), eel (thifluzamide, one case at 7 ng/g), and far eastern catfish (oxadiazon, three cases at 8–10 ng/g). No marine fish except sea bass, freshwater fish rainbow trout, whiteleg shrimp, and abalone exhibited the presence of pesticides.

The persistence of certain pesticides in water poses a substantial risk to the aquatic ecosystem, particularly for freshwater fish. Surface water contamination by pesticides used in agricultural areas adjacent to fish farms and its hazardous effects might vary depending on the degree of contamination and pesticide properties [29]. Freshwater fish exhibit a higher detection frequency compared with marine fish, possibly attributable to the environmental factors in their habitat. However, upon examining the monitoring results by species, the marine fish sea bass showed the highest frequency, with 9 out of 20 samples containing detectable levels of three pesticides, each at 10 ng/g. This is attributed to the closer geographical proximity of the aquaculture sites to land compared with that of other marine fish samples. As the detected compounds possess a log Kow value of ≥3.0, indicating their hydrophobic nature and strong bioaccumulation potential, it is imperative to conduct continuous monitoring.

### 3.3. National MRL for Detected Pesticides and Analysis of Detection Causes

Among the 44 pesticides monitored in domestic seafood samples, residue concentrations of seven detected pesticides (DDT (4,4′-DDE/4,4′-DDT), boscalid, isoprothiolane, oxadiazon, pendimethalin, thifluzamide, and trifluralin) were compared with pesticide MRL data for seafood from the United States, Australia, and Japan (Table 5). Japan has established standards for all six pesticides except boscalid, whereas the United States has set MRL for DDT (5000 ng/g for fish, edible parts) and pendimethalin (50 ng/g for freshwater crayfish). Australia has only established an MRL for DDT in fish and crustaceans (1000 ng/g) (DDE is included within the DDT standard). In Japan, the MRL for DDT is set at 1000 ng/g for fish and 3000 ng/g for crustaceans and shellfish, whereas the MRL for pendimethalin is 300 ng/g for fish. Additionally, the MRL for the pesticides isoprothiolane, oxadiazon, thifluzamide, and trifluralin are set at 3000, 600, 1000, and 500 ng/g, respectively. The residue levels of the detected pesticides in this monitoring were found to be considerably lower than foreign standards. Although South Korea currently lacks standard pesticide residue limits for seafood, all detected pesticide residue levels were below the PLS threshold of 10 ng/g.

A report on DDT monitoring in seafood indicated concentrations of 0.67, 0.79, and 1.58 ng/g dw in fat greenling, olive flounder, and fine-spotted flounder, respectively, from Asan Bay in South Korea [30]. DDT was detected in mussels from Northern Ireland at 3–30 ng/g and in those from Wales at <2–68 ng/g [31]. Despite the prohibition of DDT use in current crop cultivation, its past use has led to persistent detection in aquatic environments and organisms both domestically and internationally. Owing to its long persistence and potential for bioaccumulation through environmental and food chain pathways, contamination remains a concern.

Although monitoring results from the United States and Japan (2011–2016) reported boscalid detections ranging from ND to 100 ng/g in shrimp, oyster, and tilapia, no MRL standard exists for fish and shellfish in the US, Australia, or Japan, probably due to the limited number of tests for boscalid in seafood and the absence of detections since 2016. However, the US Geological Survey reported the presence of boscalid in 72% of samples taken from rivers, ponds, and shallow groundwater near or within farms using boscalid as a preventative fungicide in three regions across the country [32,33]. Currently, boscalid is widely used in Korea as a fungicide in the cultivation of various crops, including *Platycodon*, ginseng, and Welsh onion. Therefore, the detection of boscalid in our seafood monitoring (2 of 20 mirror carp at 8 ng/g) could be attributed to pesticides entering rivers from agricultural pesticide usage or contamination from fish feed imported from other countries [34], suggesting the possibility of contamination through feed as a potential source.

Isoprothiolane, oxadiazon, pendimethalin, and thifluzamide are used to control annual weeds during rice cultivation as well as to prevent outbreaks of rice blast and bacterial leaf blight [15,35]. Pesticides with high release rates are more likely to enter rivers and directly impact water systems [15,36]. Reports from South Korea have shown the detection of isoprothiolane, oxadiazon, and thifluzamide in rivers during the rice transplanting and farming seasons [37]. Consequently, pesticides heavily used in the cultivation of rice, the country’s staple food, are believed to have been released from rice paddies and fields, subsequently contaminating fish.

Although trifluralin has not been reported domestically or internationally since 2013, similar to other pesticides, it is presumed that this pesticide, commonly used as an herbicide in crops, could have been discharged into rivers and consequently contaminated seafood. Therefore, continuous monitoring of pesticide residues in seafood is deemed necessary to ensure safety.

### 3.4. Health Risk of Fish Consumption

The risk assessment was based on the detection results in fish products and intake data. DDT levels were assessed by combining the detected amounts of 4,4’-DDE and 4,4’-DDT. The intake of each seafood group and fish species is shown in Table 6, whereas Table 7 presents the health risk index (HI, %ADI) for the seven components detected in seafood across various scenarios.

For scenarios 1–3, the HI ranged from 0.00–0.17% ADI (with oxadiazon at 0.12–0.17). Scenarios 4–6, reflecting average consumption by fish species, showed very low HI levels ranging from 0.00–0.04% ADI. Meanwhile, scenarios 7–9 based on extreme (99th percentile) consumption, exhibited HI values between 0.00–1.07% ADI (with oxadiazon at 0.14–1.07). Overall, the HI across all scenarios indicated a very low level for all the detected pesticides. According to the guidelines provided by the Ministry of Food and Drug Safety (MFDS), a %ADI exceeding 100 is considered potentially harmful. The ADI is a measure set by regulatory agencies to determine the maximum amount of a substance that can be safely consumed on a daily basis over a person’s lifetime without causing harmful effects; it applies to intentionally used substances such as pesticides [38]. Additionally, the FAO/WHO reports that pesticide residues are considered low risk if the %ADI is less than 10% [39].

Hasan [40] reported that pesticide residue monitoring and risk assessment of fish in Bangladesh showed low risk with a %ADI of 0.01–0.02%. Consistent with these findings, the HI (%ADI) resulting from seafood consumption was below 1.07%, suggesting no potential risk to human health despite trace amounts of certain pesticides in seafood. Therefore, the consumption of farmed seafood contaminated with pesticides, even at average or extreme (99th percentile) levels, is considered highly safe. Additionally, pesticides typically undergo a reduction in residue levels during washing and processing, such as volatilization and thermal degradation, which further decreases their residual quantities [41]. Therefore, it is plausible that the health risk of the consumption of farmed seafood is even lower.

Although seven pesticides were detected in domestically farmed seafood during this monitoring study, the risk assessment demonstrated a very low risk to human health. However, due to potential variations in seafood consumption patterns influenced by consumer preferences, continuous monitoring and systematic management across the seafood sector remain necessary.

## 4. Conclusions

In this monitoring study, we analyzed pesticide residues of 44 pesticides from 15 seafood varieties collected from nine regions across the country. We evaluated their MRL for safety and potential health risks to consumers. Our findings revealed 27 cases of eight pesticides in seven types of fish out of a total of 300 samples, with a notably higher detection rate observed in freshwater species. Nonetheless, the detected pesticide concentrations were considerably below the thresholds established by both national and international standards for pesticide residues.

Our analysis suggests that agricultural pesticides may contaminate nearby water bodies and affect aquatic ecosystems over prolonged periods. Despite this, risk assessment revealed an exceptionally low health risk index (%ADI) across all scenarios, indicating a negligible health risk associated with the consumption of the investigated seafood. These results offer reassurance that continual exposure to the detected pesticide levels is unlikely to cause harmful health effects, reinforcing the safety of seafood consumption. These findings are valuable for consumers, offering assurance regarding the safety of seafood concerning pesticide residues. However, continuous and systematic pesticide monitoring and management of seafood are imperative due to the persistent, although low, risk of pesticide bioaccumulation. Further studies are warranted to explore domestic pesticide application patterns, establish specific residue limits for seafood, and investigate the efficacy of cleaning and processing techniques in decreasing pesticide residue levels. These efforts are crucial for validating seafood safety and management strategies and will ultimately aid in safeguarding public health and improving food safety.

## Figures and Tables

**Table 1:** Experimental conditions for GC–MS/MS analysis in multiple reaction monitoring mode.

Pesticide	Retention Time (min)	Precursor Ion (*m*/*z*)	Product Ion (*m*/*z*)	Collision Energy (eV)
2,4′-DDD	11.856	237	165	35
		235	165	35
2,4′-DDE	10.907	248	176	45
		246	176	45
2,4′-DDT	11.962	237	165	35
		235	165	35
4,4′-DDD	12.011	237	165	35
		235	165	30
4,4′-DDE	11.31	248	176	45
		246	176	45
4,4′-DDT	12.593	237	165	35
		235	165	35
Alachlor	9.598	237	160	10
		188	160	10
Aldrin	10.091	263	193	45
		263	191	45
Ametryn	9.577	227	185	5
		227	170	15
Atrazine	8.538	215	200	45
		215	58	25
α-HCH (α-BHC)	8.310	217	181	20
		181	145	25
β-HCH (β-BHC)	8.729	217	181	10
		181	145	20
γ-HCH (γ-BHC, Lindane)	8.730	217	181	10
		181	145	20
Boscalid	16.491	140	112	15
		140	76	35
Buprofezin	11.764	175	132	15
		172	57	15
Carfentrazone-ethyl	12.361	340	312	15
		312	151	30
Chlordane (cis)	10.884	375	266	35
		373	266	30
Chlordane (trans)	10.885	375	266	35
		373	266	35
Chlorothalonil	9.080	266	231	25
		266	170	35
Chlorpyrifos	10.106	314	258	25
		199	171	20
Cypermethrin	16.516	165	91	15
		163	127	5
Deltamethrin	18.012	253	174	10
		253	93	25
Dieldrin	11.403	265	193	45
		263	193	45
Endosulfan sulfate	12.589	272	237	20
		270	235	20
		239	204	20
α-Endosulfan	11.052	241	206	20
		205	170	20
β-Endosulfan	12.866	207	172	20
		205	170	20
Endrin	11.403	263	193	45
		263	191	45
Fenitrothion	9.841	277	260	5
		277	109	20
Heptachlor	9.648	274	237	20
		272	237	20
Heptachlor epoxide (cis)	10.638	353	253	20
		217	182	30
Heptachlor epoxide (trans)	10.639	353	253	25
		217	182	30
Hexachlorobenzene	8.419	284	249	20
		284	214	45
Iprobenfos	9.165	204	121	45
		204	91	10
Isoprothiolane	11.208	231	189	10
		189	89	25
Mirex	14.826	272	237	20
		272	143	50
		270	235	20
Oxadiazon	11.319	258	175	10
		175	112	20
Pendimethalin	10.838	194	208	5
		252	162	10
Permethrin	15.652	183	168	20
		183	155	10
Prometryn	9.616	241	199	5
		241	184	15
Tebuconazole	12.841	252	127	30
		250	125	30
Terbutryn	9.787	241	185	5
		185	170	10
Tetraconazole	10.179	336	218	25
		336	204	40
Thifluzamide	11.861	194	166	15
		194	125	35
Trifluralin	8.044	306	264	10
		264	206	5

**Table 2:** Nine scenarios for EDI.

	Daily Food Intake (DFI)		Detected Pesticide Concentration (DPC)
EDI Scenario ^a^ (9 cases)	1 (Average intake by seafood group)	×	A (Detected pesticide + LOQ value for non-detection)/number of test (20)
	2 (Average intake by fish species)		B (Detected pesticide/number of detections)
	3 (Extreme (99th percentile) intake by fish species)		C Maximum detected pesticide

^a^ EDI Scenario: combination of daily food intake (1, 2, 3) with detected pesticide concentration for the sample (A, B, C). Scenario 1 (1 × A), scenario 2 (1 × B), scenario 3 (1 × C), scenario 4 (2 × A), scenario 5 (2 × B), scenario 6 (2 × C), scenario 7 (3 × A), scenario 8 (3 × B), scenario 9 (3 × C).

**Table 3:** Linearities, limits of detection, limits of quantitation, recoveries, and precisions of 44 multiclass pesticides.

Pesticides	Linearities ^a^ (R^2^)	LOD ^b^ (ng/g)	LOQ ^c^ (ng/g)	Recovery (%)		
				LOQ	10× LOQ	50× LOQ
2,4′-DDD	0.99937	2	7	88.3–103.2	82.8–105.2	85.1–109.5
2,4′-DDE	0.99931	2	7	88.0–102.2	83.5–103.1	86.8–108.3
2,4′-DDT	0.99752	2–3	7–9	86.7–103.4	81.0–103.9	84.7–108.8
4,4′-DDD	0.99935	2	7	82.2–102.6	78.2–103.4	81.6–107.8
4,4′-DDE	0.99930	2	7	86.6–103.4	81.2–103.7	85.8–108.8
4,4′-DDT	0.99778	2–3	7–9	86.0–102.4	81.2–104.1	86.8–109.8
Alachlor	0.99792	2	7–8	82.2–103.2	78.2–105.0	79.3–110.8
Aldrin	0.99777	3	9	86.4–104.5	82.8–107.2	87.5–113.3
Ametryn	0.99946	2	7–8	73.5–103.0	70.0–106.0	70.9–113.1
Atrazine	0.99900	2	7	82.9–96.7	76.6–99.0	78.9–103.9
Boscalid	0.99695	2	7	73.3–107.2	72.5–109.1	78.0–116.4
Buprofezin	0.99741	2	7–8	74.4–103.6	71.1–105.1	71.0–110.5
Carfentrazone-ethyl	0.99788	2	7	70.5–104.4	78.7–105.8	77.6–111.2
Chlordane (cis)	0.99881	2	7	89.5–106.9	85.0–105.7	85.2–114.5
Chlordane (trans)	0.99901	2	7	86.1–106.2	86.2–108.1	87.5–114.3
Chlorothalonil	0.99934	3	10	86.9–100.3	80.9–101.5	84.9–108.1
Chlorpyrifos	0.99926	2	7	84.0–101.9	78.2–104.2	80.8–109.4
Cypermethrin	0.99565	3	10	99.4–107.4	84.9–103.4	77.4–109.2
Deltamethrin	0.99384	3	10	80.9–102.3	79.1–103.9	72.4–109.5
Dieldrin	0.99584	2–3	7–10	78.9–107.1	81.8–109.1	72.4–115.6
Endosulfan sulfate	0.99905	2	7	86.5–101.8	80.9–106.2	88.2–112.6
α-Endosulfan	0.99853	2	7	83.8–103.5	85.7–105.1	83.5–111.2
β-Endosulfan	0.99893	2	7	82.7–103.4	78.3–106.4	79.3–111.1
Endrin	0.99741	2–3	7–9	75.5–106.4	71.3–109.6	71.7–117.7
Fenitrothion	0.99793	2	7	85.8–103.2	79.2–105.1	83.2–111.3
Heptachlor	0.99911	2	7	87.2–100.8	86.4–103.4	88.4–109.0
Heptachlor epoxide (cis)	0.99743	2	7	83.2–101.6	80.3–104.2	85.7–109.4
Heptachlor epoxide (trans)	0.99863	2	7	85.6–104.0	77.0–106.4	84.5–111.6
Hexachlorobenzene	0.99936	3	10	88.8–103.9	85.1–106.2	90.9–111.9
Iprobenfos	0.99720	2	7	81.6–103.9	78.9–106.2	78.4–112.3
Isoprothiolane	0.99669	2	7	90.7–106.6	87.5–108.9	87.9–116.7
Mirex	0.99925	2–3	7–9	68.4–107.6	65.9–111.0	68.8–117.6
Oxadiazon	0.99901	2	7	84.6–106.2	80.9–108.2	84.0–114.2
Pendimethalin	0.99859	2	7	77.4–102.4	80.1–98.5	84.6–99.7
Permethrin	0.99668	3	10	82.7–103.3	75.3–105.8	90.0–111.2
Prometryn	0.99910	2–3	8–10	74.2–106.2	73.8–109.1	72.6–115.9
Tebuconazole	0.99443	3	10	78.6–115.3	105.2–116.3	110.0–117.8
Terbutryn	0.99830	2	7–8	78.3–103.8	75.2–109.7	76.2–115.8
Tetraconazole	0.99465	2	7	105.5–119.1	94.6–111.0	88.8–117.1
Thifluzamide	0.99599	2	7	100.4–103.3	86.3–106.4	85.5–114.5
Trifluralin	0.99599	2	7	87.2–109.3	83.0–111.6	86.0–117.9
α-HCH (α-BHC)	0.99912	2	7	94.4–104.3	92.1–104.9	91.4–110.2
β-HCH (β-BHC)	0.99947	2	7	97.5–102.5	82.6–104.7	87.9–108.8
γ-HCH (γ-BHC, Lindane)	0.99943	2	7	89.1–103.2	85.1–104.6	87.5–109.9

^a^ Linearities: Average of 4 fishes. ^b^ LOD, Limits of detection; ^c^ LOQ, limits of quantitation.

**Table 4:** Detection rate and residue concentration of detected pesticides from analyzed fish.

Group	Species	Sample (N)	Detected Pesticide	Detection Number	Min (ng/g)	Max (ng/g)	Mean (ng/g)
Freshwater fish	Carp	20	4,4′-DDE	1	7	7	7
	Chinese muddy loach	20	4,4′-DDE	3	7	8	7
			Pendimethalin	1	7	7	7
	Crucian carp	20	Thifluzamide	2	10	10	10
			Oxadiazon	1	7	7	7
	Eel	20	Thifluzamide	1	7	7	7
	Far eastern catfish	20	Oxadiazon	3	8	10	9
	Mirror carp	20	Boscalid	2	8	8	8
			Isoprothiolane	1	8	8	8
			Oxadiazon	2	8	10	9
			Trifluralin	1	7	7	7
	Rainbow trout	20	ND	-	-	-	-
Marine fish	Flathead mullet	20	ND	-	-	-	-
	Korean rockfish	20	ND	-	-	-	-
	Olive flounder	20	ND	-	-	-	-
	Red seabream	20	ND	-	-	-	-
	Sea bass	20	4,4′-DDE	6	10	10	10
			4,4′-DDT	2	10	10	10
			Pendimethalin	1	10	10	10
	Starry flounder	20	ND	-	-	-	-
Crustaceans	Whiteleg shrimp	20	ND	-	-	-	-
Shellfish	Abalone	20	ND	-	-	-	-
Total		300		27			

**Table 5:** Comparison of results with maximum residue limits (MRL) standard of pesticides in Japan, USA, and Australia.

Pesticides	Current Study		MRL (ng/g)		
	Fish	Mean (ng/g)	Japan	USA	Australia
Boscalid	Mirror carp	8	-	-	-
DDT	Carp	7	1000 (Fish) 3000 (Crustaceans, shelled mollusk)	5000 AL ^a^ (Fish: edible portion)	1000 E ^b^ (Fish, crustaceans)
	Chinese muddy loach	7			
	Sea bass	10			
Isoprothiolane	Mirror carp	8	3000 (Fish)		
Oxadiazon	Crucian carp	7	600 (Fish)		
	Far eastern catfish	9			
	Mirror carp	9			
Pendimethalin	Chinese muddy loach	7	300 (Fish)	50 (Crayfish)	
	Sea bass	10			
Thifluzamide	Crucian carp	10	1000 (Fish)		
	Eel	7			
Trifluralin	Mirror carp	7	500 (Fish)		

^a^ Action level. ^b^ Extraneous residue limits (ERL).

**Table 6:** Korean food consumption for 15 fish species and three groups of seafood.

Seafood		Food Consumption (g/Person/Day) in KNHANES ^a^ (2017–2021)	
		Mean	High (99th Percentile)
Fish Species			
Freshwater fish	Carp	0.4800	12.1200
	Chinese muddy loach	0.9600	43.6800
	Crucian carp	0.4800	12.1200
	Eel	1.3200	24.2400
	Far eastern catfish	0.4800	12.1200
	Mirror carp	0.4800	12.1200
	Rainbow trout	0.4800	12.1200
Marine fish	Flathead mullet	0.4800	12.1200
	Korean rockfish	1.2000	31.2000
	Olive flounder	1.3500	48.4500
	Red seabream	0.4800	12.1200
	Sea bass	0.4800	12.1200
	Starry flounder	0.4800	12.1200
Crustaceans	Whiteleg shrimp	1.8000	50.4000
Shellfish	Abalone	0.6000	13.8000
Seafood Group			
Crustaceans		3.2400	-
Fish		31.1369	-
Shellfish		4.3628	-

^a^ Korea National Health and Nutrition Examination Survey.

**Table 7:** Acceptable daily intake (ADI) values of pesticides and dietary exposure assessment for seafood.

Compound	ADI (mg/kg b.w./Day)	HI %ADI								
		Seafood Group			Fish Species					
		S1 ^a^	S2	S3	S4	S5	S6	S7	S8	S9
Boscalid	0.04	0.01	0.01	0.01	0.00	0.00	0.00	0.09	0.00	0.09
DDT	0.01	0.05	0.05	0.06	0.02	0.00	0.02	0.43	0.10	0.44
Isoprothiolane	0.1	0.01	0.00	0.01	0.00	0.00	0.00	0.04	0.00	0.04
Oxadiazon	0.0036	0.13	0.12	0.17	0.04	0.01	0.04	1.04	0.14	1.07
Pendimethalin	0.13	0.00	0.00	0.01	0.00	0.00	0.00	0.03	0.01	0.03
Thifluzamide	0.014	0.03	0.03	0.04	0.01	0.00	0.01	0.27	0.04	0.27
Trifluralin	0.015	0.03	0.02	0.03	0.01	0.00	0.01	0.25	0.01	0.25

^a^ S: scenario.

## Data Availability

The data presented in this study are available upon request from the corresponding auther due to legal restrictions.

## References

[1] Singh J., Bal J.S., Singh S., Mirza A. (2018). Assessment of chemicals and growth regulators on fruit ripening and quality: A review. Plant Arch..

[2] Afful S., Anim A.K., Serfor-Armah Y. (2010). Spectrum of organochlorine pesticide residues in fish samples from the Densu Basin. Res. J. Environ. Earth Sci..

[3] Ccanccapa A., Masiá A., Navarro-Ortega A., Picó Y., Barceló D. (2016). Pesticides in the Ebro River basin: Occurrence and risk assessment. Environ. Pollut..

[4] Hwang I.S., Oh Y.J., Kwon H.Y., Ro J.H., Kim D.B., Moon B.C., Oh M.S., Noh H.H., Park S.W., Choi G. (2019). Monitoring of pesticide residues concerned in stream water. Korean J. Environ. Agric..

[5] Albanis T.A., Hela D.G., Sakellarides T.M., Konstantinou I.K. (1998). Monitoring of pesticide residues and their metabolites in surface and underground waters of Imathia (N. Greece) by means of solid-phase extraction disks and gas chromatography. J. Chromatogr. A.

[6] Meylan W.M., Howard P.H., Boethling R.S., Aronson D., Printup H., Gouchie S. (1999). Improved method for estimating bioconcentration/bioaccumulation factor from octanol/water partition coefficient. Environ. Toxicol. Chem. Int. J..

[7] Ministry of Food and Drug Safety (2024). Korea Pesticides MRLs. http://www.foodsafetykorea.go.kr/residue/main.do.

[8] US EPA (2024). Tolerances and Exemptions for Pesticide Chemical Residues in Food, 40 CFR Part 180. http://www.ecfr.gov/cgi-bin/text-idx?SID=c1754a948c8c16c40ba981f6c2b988e6&mc=true&node=pt40.24.180&rgn=div5#se40.24.180_1493.

[9] Australian Government Agricultural and Veterinary Chemicals Code (MRL Standard) Instrument 2022. https://www.legislation.gov.au/Details/F2022C00304.

[10] European Commission (2024). EU Pesticides Database. https://food.ec.europa.eu/plants/pesticides/eu-pesticides-database_en.

[11] Ministry of Health (2024). Labour and Welfare: Maximum Residue Limits (MRLs) List of Agricultural Chemicals in Foods. https://db.ffcr.or.jp/front/.

[12] (2023). Codex Alimentarius: Codex Pesticides Residues in Food Online Database. http://www.fao.org/fao-who-codexalimentarius/codex-texts/dbs/pestres/en/.

[13] Korea Rural Economic Institute (2022). 2021 Food Balance Sheet. https://library.krei.re.kr/pyxis-api/1/digital-files/31cb7e6e-d223-4798-821a-8baf98ea55e4.

[14] Food and Agriculture Organization of the United Nations 2023—With major processing by Our World in Data. https://ourworldindata.org/grapher/fish-and-saefood-consumption-per-capita.

[15] Lee J.H., Park B.J., Kim J.K., Kim W.I., Hong S.M., Im G.J., Hong M.K. (2011). Risk assessment for aquatic organisms of pesticides detected in water phase of six major rivers in Korea. Korean J. Pestic. Sci..

[16] Choi J.Y., Yang D.B., Hong G.H., Kim S.H., Chung C.S., Kim K.R., Cho K.D. (2012). Potential human risk assessment of PCBs and OCPs in edible fish collected from the offshore of Busan. J. Korean Soc. Environ. Eng..

[17] Lim S.J., Oh Y.T., Yang J.Y., Ro J.H., Choi G.H., Ryu S.H., Moon B.C., Park B.J. (2016). Development of multi-residue analysis and monitoring of persistent organic pollutants (POPs)-Used organochlorine pesticides in Korea. Korean J. Pestic. Sci..

[18] Ministry of Environment (2021). Persistent Organic Pollutants Environmental Monitoring. http://www.me.go.kr/home/web/policy_data/read.do?menuId=10276&seq=7732.

[19] Lee K.Y. (2014). Research Planning on a Comprehensive Management Network for Risk from Environmental Pollutants.

[20] US FDA National Shellfish Sanitation Program (NSSP). https://www.fda.gov/food/federalstate-food-programs/national-shellfish-sanitation-program-nssp.

[21] National Oceanic and Atmospheric Administration (U.S. Department of Commerce) National Program Offices. https://www.fisheries.noaa.gov/contact-directory/national-program-offices.

[22] Ministry of Food and Drug Safety (2022). Veterinary Drug Positive List System for livestock and Fisheries Products. https://www.mfds.go.kr/brd/m_220/view.do?seq=32852.

[23] Ministry of Food and Drug Safety (MFDS) (2022). Food Code (N. 2021-54). https://www.mfds.go.kr/eng/brd/m_15/view.do?seq=72437&srchFr=&srchTo=&srchWord=&srchTp=&itm_seq_1=0&itm_seq_2=0&multi_itm_seq=0&company_cd=&company_nm=&page=1.

[24] Lehotay S.J. (2007). Determination of pesticide residues in foods by acetonitrile extraction and partitioning with magnesium sulfate: Collaborative study. J. AOAC Int..

[25] Food and Drug Administration (1994). Pesticide Analytical Manual. Vol1: Multi Residue Method.

[26] FAO, WHO (2023). Codex Alimentarius Commission Procedural Manual.

[27] Lammerding A.M., Fazil A. (2000). Hazard identification and exposure assessment for microbial food safety risk assessment. Int. J. Food Microbiol..

[28] (2021). KNHANES (Korea National Health and Nutrition Examination Survey). https://knhanes.kdca.go.kr/knhanes/main.do.

[29] Warren N., Allan I.J., Carter J.E., House W.A., Parker A. (2003). Pesticides and other micro-organic contaminants in freshwater sedimentary environments—A review. Appl. Geochem..

[30] Choi J.Y., Lee S.G., Bang J.H., Yang D.B., Hong G.H., Shin K.H. (2011). On the distribution of PCBs and organochlorine pesticides in fish and sediment of the Asan Bay. Ocean Polar Res..

[31] (2021). Pesticide Residues in Food, 2016–2020, Department for Environment, Food and Rural Affairs. United Kingdom. https://www.data.gov.uk/dataset/5d5028ef-9918-4ab7-8755-81f3ad06f308/pesticide-residues-in-food.

[32] Reilly T.J., Smalling K.L., Orlando J.L., Kuivila K.M. (2012). Occurrence of boscalid and other selected fungicides in surface water and groundwater in three targeted use areas in the United States. Chemosphere.

[33] USGS (2016). Fungicides from Areas of Intense Use Detected in Streams and Groundwater.

[34] Ministry of Food and Drug safety (MFDS) (2022). Preliminary Planning Research on the Safety Management of Pesticide Residues in Fishery Products. https://scienceon.kisti.re.kr/commons/util/originalView.do?cn=TRKO202300003848&dbt=TRKO&rn=&keyword=Preliminary%20planning%20research%20on%20the%20safety%20management%20of%20pesticide%20residues%20in%20Fishery%20products.

[35] (2022). Korea Crop Protection Association: Crop Protection Agent Guidelines. http://www.koreacpa.org/korea/bbs/board.php?bo_table=3_3.

[36] Kallenborn R., Burkow I.C., Schlabach M., Jørgensen E.H. (1997). PCB and pesticide distribution in cod (*Gadus morhua*), sea trout (*Salmo trutta*) and Arctic charr (*Salvelinus alpinus*) from the Norwegian Arctic. Organohalogen Compd..

[37] National Institute of Agricultural Sciences (2015). Assessment on Pesticidal Pollution of Water Systems Combined with Fate Prediction and Pesticide-Residue Monitoring.

[38] MFDS (2019). Common Guidelines for Risk Assessment of Human Applications. https://www.mfds.go.kr.

[39] Joint FAO/WHO Expert Committee on Food Additives (JECFA) (2000). Procedures for Recommending Maximum Residue Limits: Residues of Veterinary Drugs in Food.

[40] Hasan G.A., Das A.K., Satter M.A. (2022). Multi residue analysis of organochlorine pesticides in fish, milk, egg and their feed by GC-MS/MS and their impact assessment on consumers health in Bangladesh. NFS J..

[41] Kim M., Cho M., An S.E., Im M.H. (2023). Reduction effects of isoprothiolane during rice washing and cooking. Korean J. Food Preserv..

